# Quality by Design (QbD) Approach for a Nanoparticulate Imiquimod Formulation as an Investigational Medicinal Product

**DOI:** 10.3390/pharmaceutics15020514

**Published:** 2023-02-03

**Authors:** Jonas Pielenhofer, Sophie Luise Meiser, Karsten Gogoll, Anna-Maria Ciciliani, Mark Denny, Michael Klak, Berenice M. Lang, Petra Staubach, Stephan Grabbe, Hansjörg Schild, Markus P. Radsak, Hilde Spahn-Langguth, Peter Langguth

**Affiliations:** 1 Department for Biopharmaceutics and Pharmaceutical Technology, Johannes Gutenberg University Mainz, 55128 Mainz, Germany; 2 Department of Dermatology, University Medical Center, Johannes Gutenberg University Mainz, 55131 Mainz, Germany; 3 Institute for Immunology, University Medical Center, Johannes Gutenberg University Mainz, 55131 Mainz, Germany; 4 3rd Department Internal Medicine, University Medical Center, Johannes Gutenberg University Mainz, 55131 Mainz, Germany

**Keywords:** quality target product profile, critical quality attributes, GMP, nanoparticles, design of experiments, IMI-Gel

## Abstract

The present article exemplifies the application of the concept of quality by design (QbD) for the systematic development of a nanoparticulate imiquimod (IMQ) emulsion gel formulation as an investigational medicinal product (IMP) for evaluation in an academic phase-I/II clinical trial for the treatment of actinic keratosis (AK) against the comparator Aldara (EudraCT: 2015-002203-28). The design of the QbD elements of a quality target product profile (QTPP) enables the identification of the critical quality attributes (CQAs) of the drug product as the content of IMQ, the particle-size distribution, the pH, the rheological properties, the permeation rate and the chemical, physical and microbiological stability. Critical material attributes (CMAs) and critical process parameters (CPPs) are identified by using a risk-based approach in an Ishikawa diagram and in a risk-estimation matrix. In this study, the identified CPPs of the wet media ball-milling process’s milling time and milling speed are evaluated in a central composite design of experiments (DoEs) approach, revealing criticality for both factors for the resulting mean particle size, while only the milling time is significantly affecting the polydispersity. To achieve a mean particle size in the range of 300–400 nm with a minimal PdI, the optimal process conditions are found to be 650 rpm for 135 min. Validating the model reveals a good correlation between the predicted and observed values. Adequate control strategies were implemented for intermediate products as in-process controls (IPCs) and quality control (QC) tests of the identified CQAs. The IPC and QC data from 13 “IMI-Gel” batches manufactured in adherence to good manufacturing practice (GMP) reveal consistent quality with minimal batch-to-batch variability.

## 1. Introduction

Quality by design (QbD) is a modern tool aiming at a systematic development of medicines by employing statistical, analytical and risk management methodology in the design, development and manufacturing of drug products [1]. Particularly for investigational medicinal products (IMPs), quality is of high concern thank to yet-incomplete knowledge of the final toxicity and potency of the API and the safety of the product. With QbD, the quality of the product is achieved in a multistage process [2], beginning with the definition of the target product quality characteristics in a quality target product profile (QTPP), followed by the identification of critical quality attributes (CQAs) taking into account the efficacy and safety of the drug product [3,4]. In the next steps, the product design and understanding phase is initiated, enabling the identification of the critical material attributes (CMAs) of the input materials, including the drug substance and the excipients. Thereafter, the process design and understanding phase begins by identifying critical process parameters (CPPs) as sources for critical variability in the product quality being managed by establishing defined limits for the CPPs (and CMAs) within which the desired product quality is assured [4]. Afterward, a planned set of controls derived from product and process understanding is installed as a part of the control strategy. The intention is to consistently monitor process performance and the product quality by quality control testing (for more details on the quality by design approach, the reader is referred to the excellent review by Yu et al. [4] as well as to related published guidelines ICH Q8 (R2) (pharmaceutical development) [3], ICH Q9 (quality risk management) [5] and ICH Q10 (pharmaceutical quality system) [6]).

In this article, the concept of QbD is applied for the optimization of a novel nanoparticulate IMQ formulation for intra- and/or transdermal administration investigated in a phase-I/II clinical trial (EudraCT: 2015-002203-28) for the treatment of actinic keratosis (AK). The formulation concept is derived from the hypothesis that the use of IMQ *nanocrystals* leads to less-adverse events of the drug thanks to lower systemic exposure. This was observed in previous studies on the development of nanocrystalline IMQ formulations in the context of transcutaneous immunization, where nanocrystals revealed a lower permeation rate in comparison to soluble IMQ over murine skin in in vitro experiments in the Franz-diffusion cell apparatus, leading to lower systemic exposure [7]. In addition, the exclusion of isostearic acid (as contained in the comparator and approved product Aldara) from the formulation should improve the tolerability because isostearic acid is known to induce adverse skin reactions [8]. These considerations are the basis of a phase-I/II clinical trial, where the objective of the study is to evaluate whether the two IMQ formulations, Aldara and IMI-Gel (which contains nanocrystalline IMQ and *no* isostearic acid), are of comparable safety and efficacy for the treatment of AK. Furthermore, it is hypothesized that IMI-Gel might be the formulation with better tolerability.

The manufacturing of drug nanoparticles or nanocrystals has found broad application, particularly for the bioavailability enhancement of poorly water-soluble drugs such as IMQ [9]. Decreasing the drug particle size to the nanoscale increases the specific surface area, leading to an increased dissolution rate, as expressed by the Noyes–Whitney equation [10], and hence increased bioavailability [11]. In intra- and/or transdermal drug delivery, recent advances have shown that nanoparticles, including nanosuspensions or nanocrystals, serve as effective carriers of topically applied substances into the skin [12]. The underlying mechanism is a size-dependent migration and accumulation of the nanoparticles into the hair follicles, with particles around 600 nm, showing the deepest follicular penetration [13]. Once migrated into the hair follicles, nanoparticles are reported to be stored for up to 10 days in the hair follicle [14], enabling the sustained release and absorption of the drug substance(s) into the skin [12,15].

In order to deliver IMQ into the hair follicles, the breakup of the initial micron-size particles (*d*_10_ 4.96 µm, *d*_50_ 13.02 µm, *d*_90_ 26.39 µm) to the nanoscale is required. From the available techniques for the preparation of drug nanoparticles, the top-down methods of wet media milling, including wet stirred media milling, planetary milling and ball milling, are most frequently used [16]. In wet media milling, drug nanoparticles are prepared by dispersing drug particles in aqueous polymeric/surfactant stabilizer solution, together with grinding beads. During milling, the coarse micron-size particles are repeatedly stressed when captured between the colliding beads, over time leading to a breakdown of the drug particles to the nanoscale, yielding a nanosuspension [16,17]. Throughout processing and storage, the nanoparticles may aggregate, forming large clusters; show surface amorphization, which would lead to the immediate dissolution of the amorphous part and the recrystallization onto the present drug crystals; or show Ostwald ripening, leading to a loss of the benefits of the large surface area and an inability to exhibit the targeted product performance [16,18]. The preparation of physically stable drug nanoparticles via wet media milling at the targeted size entails (i) the selection and optimization of effective equipment process parameters, (ii) the consideration of the physicochemical and mechanical properties of the drug substance and (iii) the selection of a suitable polymeric/surfactant stabilizer [18]. With respect to IMQ nanocrystal preparation, the evaluation and selection of appropriate process parameters and stabilizing excipient(s) to produce physically stable drug nanoparticles at the targeted size is required to generate a high-quality pharmaceutical product for AK patients [18,19].

To achieve this objective, the critical process parameters (CPPs) of the milling time and the rotational speed, identified in an Ishikawa diagram as a risk-based approach, are evaluated and optimized by applying the concept of the design of experiments (DoEs) in a face-centered central composite design (CCD). The target particle size and polydispersity are identified on the basis of a designed quality target product profile (QTPP) outlining the biopharmaceutically critical quality attributes (CQAs) that are most relevant for the IMP used for the treatment of AK patients in the respective phase-I/II clinical trial. Moreover, the critical factors from the input materials affecting the target product quality referred (critical material attributes (CMAs)) to in previously conducted studies are discussed. In addition, the installation of appropriate controls during manufacturing and for batch release testing are put in place. Additionally, quality metric data from manufactured and released batches are presented.

## 2. Materials and Methods

### 2.1. Materials

IMQ drug substance of GMP grade was purchased from Teva Pharmaceuticals Industries Ltd.—Teva API Division (Debrecen, Hungary). Aponorm 7 mL tubes, Aqua conservata DAC, Carbopol 974P, jojoba wax, polysorbate 80 and sodium hydroxide were purchased from Caesar & Loretz GmbH (Hillscheid, Germany). Zirconium oxide milling spheres of 1 mm diameter were purchased from Fritsch GmbH (Idar-Oberstein, Germany). Acetonitrile ≥ 99.9%, HiPerSolv CHROMANORM gradient grade and methanol ≥ 99.8%, HiPerSolv CHROMANORM gradient grade, WTW technical buffers TEP 2, STP-7 and STP-10 trace were purchased from VWR International GmbH (Darmstadt, Germany). Phosphoric acid LiChropur > 85%, sulfuric acid ≥ 98% LiChropur, Millex nylon syringe filters of 0.45 µm pore size and a diameter of 25 mm, triethylamine ≥ 99.5% LiChropur and IMQ USP Reference Standard were purchased from Sigma Aldrich Chemie GmbH (Taufkirchen, Germany). Ethanol Rotisolv HPLC gradient grade, heptane-1-sulphonic acid sodium salt, sodium lauryl sulfate ≥ 99% and Omnifix disposable 50 mL syringes with Luer-Lock fitting were purchased from Carl Roth GmbH (Karlsruhe, Germany). Inertsil ODS3-5 µm 250 mm × 4.6 mm and Zorbax RX-C8 150 mm × 4.6 mm 5 µm HPLC columns were purchased from MZ Analysentechnik GmbH (Mainz, Germany). 

### 2.2. Design of the QTPP and Identification of CQAs

As a basis for the QTPP, the product characteristics of the comparator product Aldara were compared against the ICH Q8 R(2) guideline on pharmaceutical development [3], and a recent review [20] was used. The target dosage form, the route of administration and the dosage strength were defined on the basis of the disease intended to be treated and the properties of the comparator product Aldara. The target stability was defined on the basis of the required time period from manufacturing, quality control testing, batch releasing, delivery to the study site and treatment period with optional extension and additional stability testing, if required. The drug product quality attributes were defined to meet the applicable standards in terms of physical attributes, including the IMQ drug particle size, rheological properties, disperse jojoba wax droplet size and pH of the formulation, as well as for the identification, the assay, the homogeneity of the formulation and tube uniformity of filled units, the limits for the impurities, the degradation products, the content for the preservatives and the microbiological limits. The container closure system and the integrity thereof were included, aiming at ensuring appropriateness for the dosage form to ensure the target shelf life.

The CQAs were identified on the basis of the projected severity of harm to the patient in case the attribute were to fall outside of the targeted range.

### 2.3. Product Design

The product is designed to meet the patients’ needs in terms of a safe and easily administrable formulation. For the treatment of AK, it is required to transport IMQ toward the immunocompetent cells in the epidermis and dermis to activate the innate and acquired immune system, ultimately leading to the apoptosis of the atypic keratinocytes that cause the clinical presentation of AK [21].

### 2.4. Process Design, Identification and Evaluation of CMAs and CPPs

*Preparation of the aqueous nanosuspension*: to create an IMQ nanosuspension, the top-down method of a wet media ball-milling process was selected. CMAs and CPPs were identified by using an Ishikawa diagram and estimated for their criticality in a risk-estimation matrix. The CMAs have been previously studied by Gogoll [7,22] and Denny [23]. In this study, the identified CPPs of the wet media ball-milling process’s milling time and rotational speed were evaluated by using DoE in a face-centered CCD and were set up by using Minitab Statistical Software (version 21.1). The CPPs were studied at two levels: low (−1) at 250 rpm for 60 min and high (+1) at 650 rpm for 240 min in 2 replicates with an alpha of 1. Milling time was segmented into 20 min cycles, each cycle with a 10 min pause interval in between each cycle, using a Fritsch Pulverisette 6 planetary mill. For the experimental runs with 150 min of milling time, a 10 min cycle was added after the seventh milling cycle. All experimental runs were randomized. The model contained 8 corner points, 10 center points and 8 star points, totaling 26 conducted experimental runs. For each experimental run, the z-average and PdI were measured in triplicates. The ratio of milling balls to drug substance mass, the media volume and the milling ball size were kept constant by following [7,22]. For the study and manufacturing of IMPs, a total mass of 25 g of 1 mm diameter zirconium oxide milling balls, a volume of 17 mL of a 9% (*w*/*w*) polysorbate 80 solution and an IMQ mass of 3 g were added into a 45 mL zirconium oxide grinding vessel [23]. Based on the identified optimal process conditions, milling for IMP manufacturing was performed at 650 rpm for 7 20 min repetitive cycles, each with a 10 min pause interval in between each milling cycle.

*Homogenization of the o/w emulsion*: for the dispersion of jojoba wax in the aqueous IMQ suspension, a high-pressure homogenization (HPH) process was selected. The number of cycles and pressure necessary to disperse the jojoba wax were identified as a critical process parameter and empirically determined until the emulsion appeared homogeneously. For the manufacturing of IMPs, the emulsion was homogenized for 5 cycles at 500 bar and 10 cycles at 1000 bar after the addition of stochiometric amounts of jojoba wax, Aqua conservata DAC and polysorbate 80 using an Avestin Emulsiflex C3.

*Adjustment of the cream consistency*: to create a cream formulation, a neutralized 3% (*w*/*w*) Carbomer 974P gel was prepared (pH 6.5–7.0). After gelling, a stochiometric amount of the neutralized carbomer gel was added to the homogenized o/w emulsion after the HPH step. The emulsion was incorporated into the gel matrix in a bowl with a pestle and mixed until the gel had transitioned from a sol to the gel state, and the formulation appeared as a cream. The resulting formulation was packaged in a 7 mL Aponorm aluminum tube with 3 g ± 15% of the formulation and labeled according to regulatory requirements on the labeling of IMPs.

#### Dynamic Scanning Calorimetry (DSC) Measurements of Milled Suspension

The melting point of the as-received IMQ crystals and that of dried IMQ nanosuspension (milled for 240 min at 650 rpm without stabilizer) were determined by using a Mettler Toledo DSC 1 (Mettler Toledo AG, Analytical, Schwerzenbach, Switzerland). A sample of 2–3 mg of powder was placed in a sealed perforated aluminum pan. The samples were heated under nitrogen flow at between 200 °C and 335 °C at a rate of 5 °C/min.

### 2.5. Manufacturing Process of the IMP IMI-Gel, Based on the Process Design and CPPs

**Figure 1:**
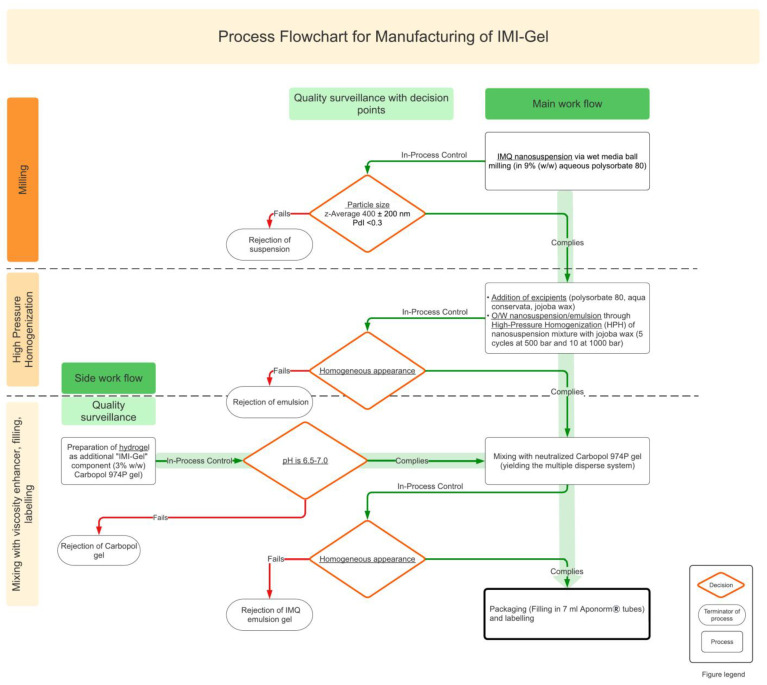
Process flowchart for manufacturing of IMI-Gel. IMQ nanocrystals are manufactured in a wet media ball-milling process, followed by an in-process-control of the resulting mean particle size and the polydispersity index. If both parameters comply with the acceptance criteria of a z-average of 400 ± 200 nm and a PdI < 0.3, the jojoba wax phase (together with the remaining stochiometric amounts of Aqua conservata DAC and polysorbate 80) are added and homogenized to fine-disperse the nanoparticle emulsion. If the formulation appears homogeneous, a stochiometric amount of a precisely neutralized Carbopol 974P gel (pH 6.5–7.0) is added and mixed with the emulsion. If the IPC of the homogeneous appearance of the formulation complies, the formulation is transferred into 7 mL Aponorm tubes.

Figure 1 reveals the manufacturing process of the IMP IMI-Gel as described in Section 2.4. 

### 2.6. Control Strategy

#### 2.6.1. Particle-Size Distribution Analysis

The particle-size distribution of the IMQ nanocrystals was measured via dynamic light scattering (DLS) using a Malvern Zetasizer Nano ZS (Malvern Panalytical Ltd., Malvern, UK) as an IPC after the manufacturing process. Three samples of 2.8 µL of the IMQ suspension were withdrawn from the grinding vessel, diluted with 4.99 mL of MilliQ water at 25 °C and measured in three consecutive runs so that 18 data points were obtained per batch. *Acceptance criterion*: z-average 400 ± 200 nm, PdI < 0.3.

#### 2.6.2. Homogeneity of the Formulation

Homogeneity of the formulation was visually assessed by inspecting the emulsion after HPH for indicators of inhomogeneity, e.g., phase separation. *Acceptance criterion*: homogeneous appearance.

#### 2.6.3. Weighing of Filled Tubes

All filled units of each manufactured batch were weighed by placing each unit without a label onto an analytical balance Precisa XR205SM-DR (Dietikon, Switzerland). *Acceptance criterion*: 5 g ± 15%.

#### 2.6.4. HPLC Assay 1: Therapeutically Active Ingredient IMQ

The content of the drug substance was analyzed per High performance liquid chromatography (HPLC)-UV using a Jasco HPLC equipped with a LC-Net II/ADC interface box, an Autosampler AS-950, a PU-980 pump, an UV/VIS UV-975 detector, a DG980-50 degasser, a LG-980-50 mixer and a Jet Stream ATP-CHY 501 column oven. ChromNav CFR 2 software was used to integrate and analyze the generated peaks of the chromatograms. As the analytical method, the method from the USP Monograph Imiquimod Cream Assay was used [24] (for details, see S1). *Acceptance criterion* 90% ≤ × ≤ 110% of labeled amount.

#### 2.6.5. HPLC Assay 2: Content of Preservatives (Methyl- and Propylparaben)

For the analysis of the content of preservatives, a previously published method was used, with some slight modifications [25] (for details, see S2). For analysis, the same system as under Section 2.6.4. was used. *Acceptance criterion*: total preservative content of 0.04–0.06% (*w*/*w*).

#### 2.6.6. HPLC Assay 3: Impurities and Degradants of IMQ

As the assay for the impurities, the method as described in the Pending USP Monograph IMQ Cream under the section “Impurities” was used [24]. For analysis, the same system as under Section 2.6.4. was used (see S3 for details). *Acceptance criteria*: impurity levels for the 5 major impurities not more than 0.2%; for unknown impurities, not more than 0.1%; and for the total amount of impurities, not more than 0.5%.

#### 2.6.7. Jojoba Wax Droplet Size

The jojoba wax droplet size was assessed by using a Leica DM IL (Wetzlar, Germany) inverted microscope at 400× magnification using a Hund Wetzlar 40/0.60-LD objective. One filled tube per batch was separated into three 750 mg samples. From these samples, one sample, per 750 mg sample, of 250 mg weight was transferred into a 50 mL volumetric flask and diluted with MilliQ water up to the mark. The samples were diluted at a 1 to 10 ratio. Here, 50 µL of each sample was placed on microscopic slides, from which 3 photos per sample were recorded. *Acceptance criterion*: oil droplet diameter of <10 µm for all droplets.

#### 2.6.8. The pH Measurement of the Final Formulation

The pH of the formulation was measured on the basis of the method described in USP 791, stating “where approximate pH values suffice, indicators and test papers may be suitable” [26]. The pH indicator papers were considered suitable for pH analysis and approved upon regulatory submission from the federal authority. The pH of the formulations was assessed by using nonbleeding pH indicator strips from pH 4.0–7.0 (4.0–4.4–4.7–5.0–5.3–5.5–5.8–6.1–6.5–7.0). Three samples per batch were measured. *Acceptance criterion*: pH of final formulation = 4.0–6.0.

#### 2.6.9. Microbiological Testing According to Ph.Eur. 2.6.12. with Acceptance Criteria according to Ph.Eur. 5.1.4.

Microbiological testing was externally performed at BioChem GmbH (Karlsruhe, Germany). Samples for microbiological quality testing were transported under controlled conditions to Karlsruhe and handed over to BioChem in person. As the test method, the pour plate method as described under Ph.Eur. 2.6.12. with acceptance criteria from Ph.Eur. 5.1.4. was used. *Acceptance criteria*: limits as defined in the Ph.Eur. 5.1.4. monograph.

### 2.7. Statistical Data Analysis and Generation of Graphs

The design of experiments (DoE) experimental plan was created and analyzed by using Minitab Statistical Software (version 21.1). The response-surface plots and DSC graphs were plotted using OriginPro 2023. The Ishikawa fishbone diagram and process flowchart were plotted using Lucidchart 2023. The HPLC chromatograms and peaks were integrated using ChromNav2 CFR software (version 2.02.08). The peaks were analyzed using Microsoft Excel 2019. The QC graphs were plotted using GraphPad Prism 9. Descriptive statistics for the observed QC data were calculated using GraphPad Prism 9.

## 3. Results

### 3.1. Definition of QTPP and Identification of CQAs

Table 1 revels the quality targeted product profile (QTPP) with the identification of the CQAs of a nanoparticulate emulsion gel formulation for the product IMI-Gel. The particle size of IMQ is identified as a crucial CQA thanks to a size-dependent follicular migration of nanoparticles [12,27].

Additional CQAs are identified on the basis of their relevance for the safety and efficacy of the formulation. For example, the rheological properties are known to be relevant for the residence time of the formulation on the skin (for ensuring suitable drug release, as indicated by the in vitro release profile relevant for the sustained absorption of IMQ in the skin). For maintaining the stability over the shelf life, the pH of the formulation is crucial for the prevention of the pH-dependent solubilization of IMQ [27]. The pH is also relevant for ensuring the efficacy of the preservatives, which are the prerequisites for microbiological quality, as the outer continuous phase of the formulation is water. Furthermore, the pH is maintaining the gel structure of the gelling agent. Also critical for efficacy/tolerability are the content of IMQ and the impurities/degradants.

### 3.2. Product Design

#### 3.2.1. IMQ Drug Substance: Considerations Regarding the Physicochemical Properties of IMQ and Nanocrystals

IMQ is a small molecule with a molecular weight of 240.30 Da and a pKa of 7.3. Its physicochemical properties make IMQ a suitable candidate for being formulated as a nanosuspension because IMQ neither dissolves in water nor changes its polymorphic form upon processing, which might alter the dissolution rate and lead to instabilities in nanosuspensions (see DSC graphs in Figure S1).

#### 3.2.2. Stabilizing Excipients for IMQ Nanocrystals and Disperse Oil Phase

For IMI-Gel, different stabilizers to prevent Ostwald ripening were considered as suitable, including the surfactant polysorbate 80 and crystal growth inhibitors PVP K30 and PVP K90. It was found that the addition of polysorbate 80 leads to the appropriate stabilization of the IMQ nanocrystals, whereas the crystal growth inhibitors PVP K30 and PVP K90 do not positively influence the stability of IMQ nanocrystals but instead lead to the fast sedimentation of IMQ particles when PVP K90 is used [22]. Given the fine dispersion of jojoba wax, polysorbate 80 is able to stabilize the dispersed jojoba wax droplets, leading to a fine emulsion.

#### 3.2.3. Selection of the Oil Component

As potential oil components, different pharmaceutically acceptable oils (including middle chain triglycerides (MCT), jojoba wax, squalene and oleic acid) were considered. A major decision criterion for selection was their influence on IMQ permeation behavior across murine skin. Among the tested oils, jojoba wax led to the strongest deceleration of IMQ permeation and was hence selected as the oil component [22].

#### 3.2.4. Gelling Agent and Neutralizing Agent

As the thickening agent, Carbopol is selected thanks to its ability to form optically pleasant hydrogels at low concentrations of 0.5–1% (*w*/*w*) upon neutralization with inorganic or organic bases. As a suitable gelling agent, it provides structural stability over a broad pH range of 4–11, where the viscosity is largely independent of the pH [29].

#### 3.2.5. Preservatives and Antioxidants

Preservatives, which have their optimal efficacy in the strong acidic pH ranges, are not suitable for IMI-Gel because this might lead to the solubilization of the nanocrystals and collapse of the carbomer gel. Furthermore, ionic preservatives affecting the Carbopol stability are excluded. Parabens (methyl- and propylparaben) show efficacy over the pH range in which Carbopol forms and maintains its structure and IMQ nanocrystals are not solubilized (from 4–8). Hence, they are selected as the preservatives.

### 3.3. Process Design and Identification of CMAs and CPPs

#### 3.3.1. Process Flowchart

Figure 1 shows the process design in form of a process flowchart for the manufacturing of IMI-Gel. For IMI-Gel, a top-down wet media ball-milling process is selected, followed by a HPH unit operation to finely disperse the oil phase. A semisolid formulation is then created by the addition of a precisely neutralized carbomer gel.

#### 3.3.2. Ishikawa Diagram and Risk-Estimation Matrix (REM) for IMI-Gel

**Figure 2:**
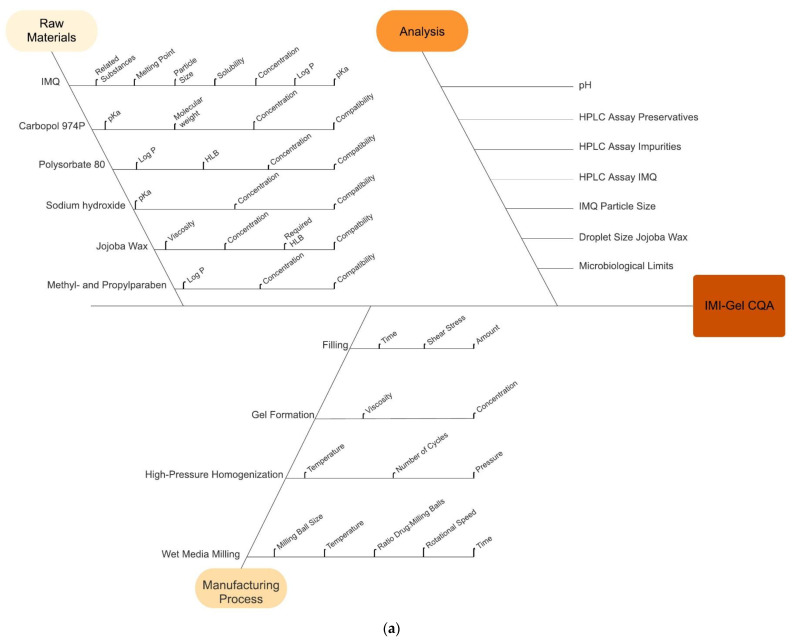

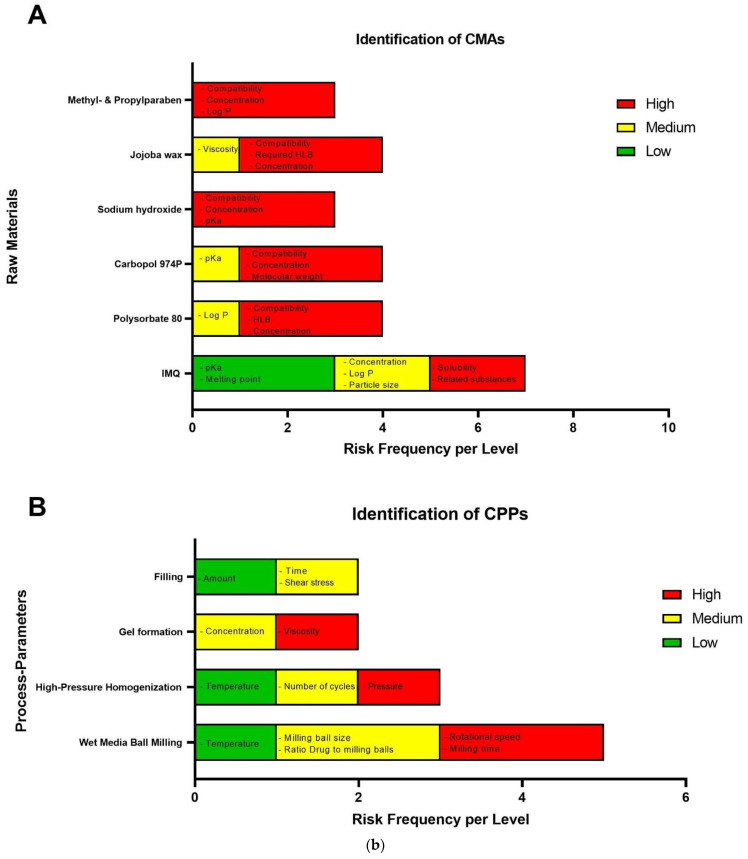
(**a**) Ishikawa diagram for the IMP IMI-Gel showing the critical parameters affecting the quality of the IMP related to the raw materials, the manufacturing process and the analytical characterization. Additional factors not depicted here are “Environment” and “People”. (**b**) Frequency histogram for identification of critical material attributes (CMAs) (**A**) and critical process parameters (CPPs) (**B**) for the IMP IMI-Gel showing the critical parameters affecting the quality of the IMP related to the raw materials and for manufacturing. CMAs and CPPs are categorized as low (in green), medium (yellow) and high (red).

In risk assessment, factors affecting the CQAs for IMI-Gel from the input materials and during manufacturing are identified by using an Ishikawa diagram and evaluated for their criticality in a risk-estimation matrix. Figure 2a depicts the Ishikawa diagram for IMI-Gel. Figure 2b depicts the major results out of the respective risk-estimation matrix for IMI-Gel (for the original matrix, see Table S1). Table 2 lists the identified CMAs.

### 3.4. Considerations and Evaluation of Selected Potential CMAs

#### 3.4.1. IMQ Drug Substance

For the IMQ drug substance, critical material attributes identified on the basis of the REM (Figure 2b and Table S1) and Ishikawa diagram (Figure 2a) are the IMQ solubility and the occurrence of related substances. Previously conducted chemical stability investigations into the criticality on the occurrence of related substances revealed no detectable levels of related substances over 6 months of storage [23].

#### 3.4.2. Stabilizing Excipients for IMQ Drug Substance

The identified CMAs for the surfactant are the compatibility between the surfactant and the drug substance, the concentration of the surfactant and the hydrophilic–lipophilic balance (HLB).

During formulation development, it was found that polysorbate 80 at a concentration of 0.9% (*w*/*w*) enabled the sufficient wettability of IMQ for the preparation of nanosuspensions [22]. Stable nanosuspensions manufactured with 0.9% (*w*/*w*) polysorbate 80 show the following characteristics: (a) a negative zeta potential of −33.1 ± 0.93 mV; (b) a size scale range of 100–500 nm, measured per scanning electron microscopy using Martins’ diameter; and (c) a mean hydrodynamic size of 285 nm, measured per DLS. The stability testing of the nanosuspension over 9 months revealed no change in the mean particle size (z-average) of the IMQ nanocrystals at the tested 0.9% (*w*/*w*) concentration [22].

#### 3.4.3. Oil Component

As CMAs for the oil component, three parameters are identified: (a) the compatibility of the formulation, (b) the concentration and (c) the required HLB (which should be met by the selected surfactant in order to achieve the ideal dispersion of the oil phase). For the emulsification of 40% (*w*/*w*) jojoba wax as the disperse phase, the required HLB is 12.5 [30] at a required final surfactant concentration of ≥4% (*w*/*w*) in the system [30]. Given the appropriate stabilization of the IMQ nanoparticles, polysorbate 80 was evaluated as the only surfactant for the emulsification of jojoba wax. For the highest impact of the deceleration of IMQ, a high concentration of jojoba wax (45%) was selected. The concentration of polysorbate 80 was selected to be 5% (*w*/*w*) on the basis of the reported ≥ 4% polysorbate 80 required for the emulsification of 40% (*w*/*w*) [30], plus the tested 0.9% (*w*/*w*) of polysorbate 80 for the stabilization of the IMQ nanocrystals. The evaluation of the compatibility revealed compatibility between the surfactant oil and IMQ nanocrystals at the given polysorbate 80 concentration (at HLB 15) and at a jojoba wax concentration of 45% (*w*/*w*) [22,23].

#### 3.4.4. Gelling Agent and Neutralizing Agent

As depicted in the REM, the CMAs of the gelling agent are identified as its concentration, its molecular weight, the target pH of the formulation thanks to the pH-dependent gel formation of Carbopol hydrogels (after neutralization) and its compatibility with IMQ. From the available Carbopol types, Carbopol 974P (medium range molar mass of 35,000 Da) is selected thanks to its ability to form hydrogels with a plastic flow behavior of a high viscosity of ~30,000 cP at pH 7.5 and at a concentration of 0.5% (*w*/*w*) [31].

For neutralization, sodium hydroxide is selected. As a CMA, its concentration is identified because it influences the final pH of the formulation (which is controlled through the addition of stochiometric NaOH and by monitoring the final pH).

#### 3.4.5. Preservatives and Antioxidants

Methyl- and propylparaben are selected as the preservatives thanks to their optimal activity between pH 4 and pH 8, which matches the targeted pH range of the formulation [32]. The effect of different methyl- and propyl-4-hydroxybenzoate concentrations on the inhibition of microbiological growth was evaluated in a conservation load test, according to Ph.Eur. 5.1.3. There, no difference at the tested concentrations ranging from 0.04% (*w*/*w*) to 1.5% (*w*/*w*) was observed [23]. All tested concentrations inhibited the microbiological growth of the inoculated microorganisms with a partial reduction in the total number of inoculated microorganisms. The required log reduction number of viable microorganisms defined in the Ph.Eur.5.1.3 was not met. The evaluation of the microbiological examination of nonsterile products specified in Ph.Eur. 2.6.12. for formulations stored over 6 months under intermediate storage conditions at 30 °C/65% RH, according to ICH Q 1 A (R2) [33], revealed that the Ph.Eur. criteria for all tested concentrations were met.

The addition of antioxidants is considered unnecessary because jojoba wax as a material contains natural antioxidants [32], hence showing high oxidative stability.

### 3.5. Evaluation of Potential CPPs

#### 3.5.1. Design of Experiments

Table 3 lists the identified most critical process parameters (CPPs) affecting the IMQ particle-size distribution during the wet media ball milling of IMQ (depicted in red in the REM and frequency histogram) and the homogeneity of the formulation after the addition of the jojoba wax phase.

In wet media milling, the frequently studied process parameters significantly affecting the breakage rate of the drug particles are milling time, milling medium (most importantly bead size and the amount of milling medium, e.g., number of beads), milling speed, drug amount and milling design [18,34,35,36]. In the current study, the effect of factors such as (a) the rotational speed (X_1_) and (b) the milling time (X_2_) on the following CQAs are studied during wet media ball milling: (I) the particle-size distribution measured as the responses of the intensity-weighted mean hydrodynamic particle size (z-average [d.nm]) and (II) the polydispersity index. A face-centered CCD is applied to determine the optimal process parameters for a mean particle size in the range of 300–400 nm and with a minimal PdI. Other parameters, including drug amount, type and amount of milling medium, bead size and milling design, are taken as previously described [22,23]. The response data for all 26 conducted experimental runs are presented in Table A1.

The statistical significance of the linear and quadratic impacts of the studied factors and their interactions with the responses as calculated by analysis of variance (ANOVA) are presented in Table 4.

The ANOVA table for the z-average reveals that linear, quadratic and two-factor interactions (2FI), except for the squared milling time, are *statistically significant* for the model parameters, with *p*-values of <0.05. An analysis of the model fit reveals an acceptable correlation between the predicted and observed values, as expressed by the regression coefficient (with the corrected regression coefficient of 0.9221 and the predicted regression coefficient of 0.8732 and an *insignificant* lack of fit value with a *p* of 0.193 (see Table 6)).

The analysis of the model yields the following regression equation:

Particle size = 837.4 − 0.996·X_1_ − 1.734·X_2_ + 0.000656·(X_1_)^2^ + 0.001901·(X_2_)^2^ + 0.000904·X_1_·X_2_ (1)

where X_1_ = rotational speed and X_2_ = milling time.

When for the PdI model, a Box-Cox transformation at a λ of 1 is conducted, the ANOVA table for the linear, quadratic and 2FI reveals *statistical significance* for the linear factor of the milling time (X_2_) with a *p* < 0.05 (see Table 4). *Statistical insignificance* is observed for the linear term for rotational speed (X_1_) with a *p* of 0.655; the quadratic terms for the rotational speed (X_1_)^2^ with a *p* of 0.835; the milling time (X_2_)^2^ with a *p* of 0.522; and the 2FI (X_1_X_2_) with a *p* of 0.690. The reduction in the model by the removal of the *insignificant* quadratic terms of the rotational speed (X_1_)^2^, the milling time (X_2_)^2^ and the 2FI (X_1_X_2_) (see Table 5) to improve the accuracy of the predicted values reveals an improved fit of the model, indicated by a reduced difference between the R-squared adjusted and R-squared predicted values (see Table 6). The linear term for the rotational speed (X_1_) is not removed from the model, because its removal would not increase the prediction accuracy to a significant extent.

The analysis of the model yields the following regression equation:

PdI = 0.3003 − 0.000018·X_1_ − 0.000618·X_2_ (2)

where X_1_ = rotational speed and X_2_ = milling time.

**Table 6:** Summary of the model fit for the studied response variables of the mean particle size (z-average [d.nm]) of the IMQ nanocrystals and for the polydispersity index (PdI) with the complete and the reduced model.

Responses	Standard Deviation S	R-Squared	R-Squared Adjusted	R-Squared Predicted
z-average [d.nm] PdI (full factors) PdI (reduced factors)	17.9720 0.0280 0.0267	0.9376 0.7073 0.6950	0.9221 0.6341 0.6685	0.8732 0.3522 0.5679

**Figure 3:**
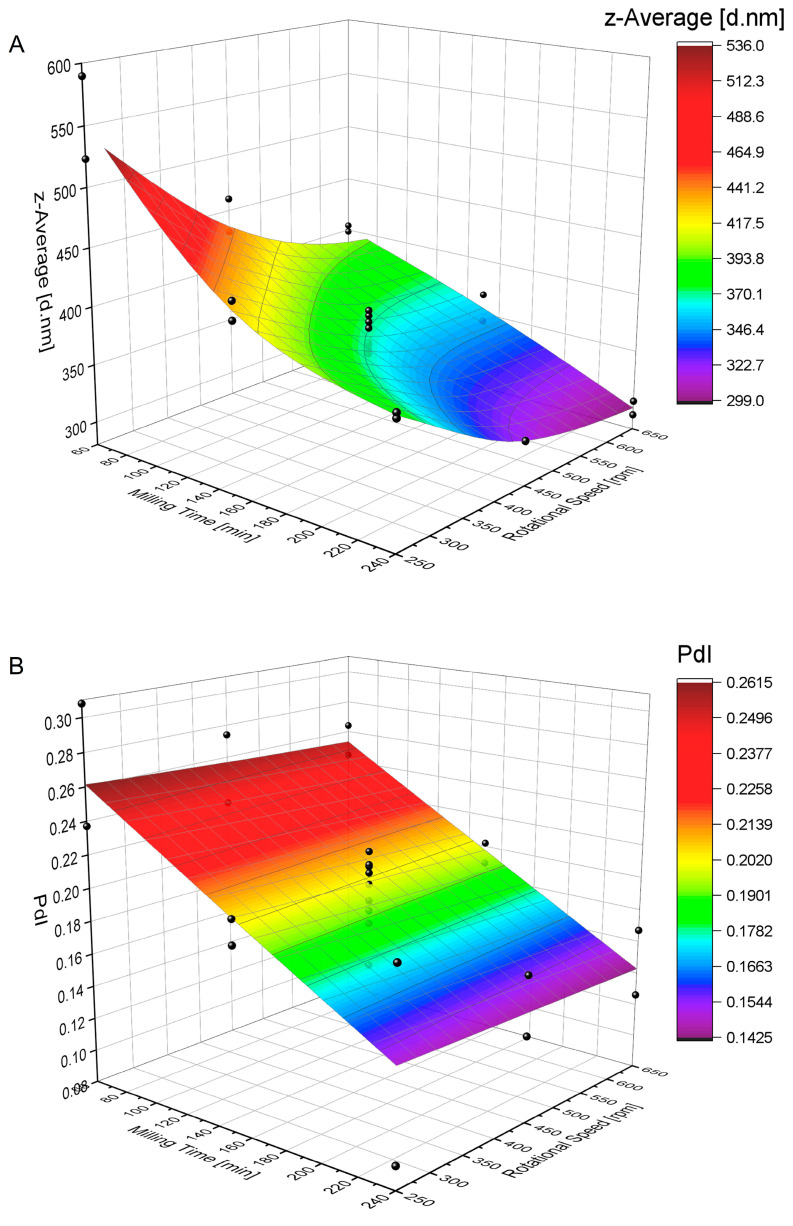
Response-surface plots with experimental points in black, illustrating the effect of the milling time and rotational speed on the z-average (**A**) and the PdI (**B**) (for optimized parameters, see Table 7).

Figure 3 visualizes the effect of the milling time and rotational speed on the mean particle size (z-average [d.nm]) and the polydispersity index. The response-surface plot for the reduction in the mean particle size in Figure 3A reveals a curvature, indicating a *nonlinear* relationship between particle-size reduction and the studied factors of the milling time and rotational speed approaching a lower limit of ~300 nm. The lowest mean hydrodynamic particle size is observed at the highest rotational speed and the longest milling time, with a z-average of 292.8 nm (run 11) and 305.2 nm (run 17), as shown in Table A1. 

Figure 3B reveals that a reduction in the PdI follows a *linear* model, where the minimal achievable PdI is a function of time.

#### 3.5.2. Validation of the Model

The purpose of validation is to evaluate the accuracy of the predictability of the model and to find optimal process conditions to achieve a mean particle size in the range of 300–400 nm with a minimal PdI (at least smaller than 0.3) at a reasonable milling time for manufacturing under GMP conditions. To evaluate these conditions, a milling time range of 80–140 min, with a target particle size of 350 nm and a minimal PdI with starting conditions of 650 rpm and 140 min, is chosen. The predicted solution is a rotational speed at 650 rpm for 135 min, resulting in a mean particle size (z-average) of 349.99 nm and a PdI of 0.205. The optimal conditions with the observed and predicted values are given in Table 7. Because the milling process was segmented into 20 min cycles, the factors were chosen to be 650 rpm and 140 min of milling time.

**Table 7:** Result from QbD approach: Optimized parameters with predicted and observed values of responses.

Factors		Predicted Values		Observed Values	
Rotational Speed [rpm]	Milling Time [min]	z-Average [d.nm]	PdI	z-Average [d.nm]	PdI
650	135	349.99	0.205	378.8 ± 11.22	0.195 ± 0.03

An analysis of the particle size reveals that with the conditions of 650 rpm and 140 min, a slightly larger mean hydrodynamic particle size is observed, with a difference of 8.23% ± 3.22% and a difference for the PdI of 4.88% ± 0.034%.

#### 3.5.3. High-Pressure Homogenization

The number of cycles to achieve a homogeneous emulsion was empirically determined until the emulsion appeared homogeneous because the droplet size was not identified as a CQA. During evaluation, it was found that with 5 cycles at 500 bar and 10 cycles at 1000 bar led to a homogeneous formulation.

### 3.6. Formulation Performance Testing

For long-term performance testing, formulations containing 45% (*w*/*w*) jojoba wax, 5% (*w*/*w*) polysorbate 80 and 5% (*w*/*w*) IMQ nanocrystals preserved with 0.045% methyl- and propylparaben were used. Formulations were stored over 6 months under intermediate storage conditions, according to ICH Q 1 A (R2), and evaluated for their chemical stability. There, it was observed that the mean IMQ content of the formulation varies from 99.85% to 100.08% with no detectable impurities, revealing chemical stability [23].

The evaluation of the microbiological quality, according to Ph.Eur. 2.6.12., in an unopened primary packaging over 6 months as well as in an in-use stability test over 2 and 4 weeks simulating potential microbiological contamination revealed no microbiological contamination, fulfilling the acceptance criteria of the Ph.Eur.

The evaluation of the formulation pH over the storage period revealed no change in pH from the targeted pH range of 4–6 over the storage period [23].

The rheological characterization of the formulation with 0.5% (*w*/*w*) Carbopol 974P revealed plastic flow behavior in the formulations and an approximately 20-fold increase in shear stress at similar shear rates for the Carbopol-based formulation when compared to the reference product Aldara (thin, fluid consistency) [22]. An assessment of the structural stability for IMI-Gel assessed in a three-interval step test for formulations stored for 12 months at ICH Q1 A (R2) under long-term conditions (25 °C/65% rH) and over 6 months under accelerated storage conditions (40 °C/75% rH) revealed structural stability for formulations precisely adjusted to a pH range of 4–6 (see [37] for the study).

### 3.7. Control Strategy

#### Data of In-Process Control and Quality Control Tests from Manufactured Batches

**Figure 4:**
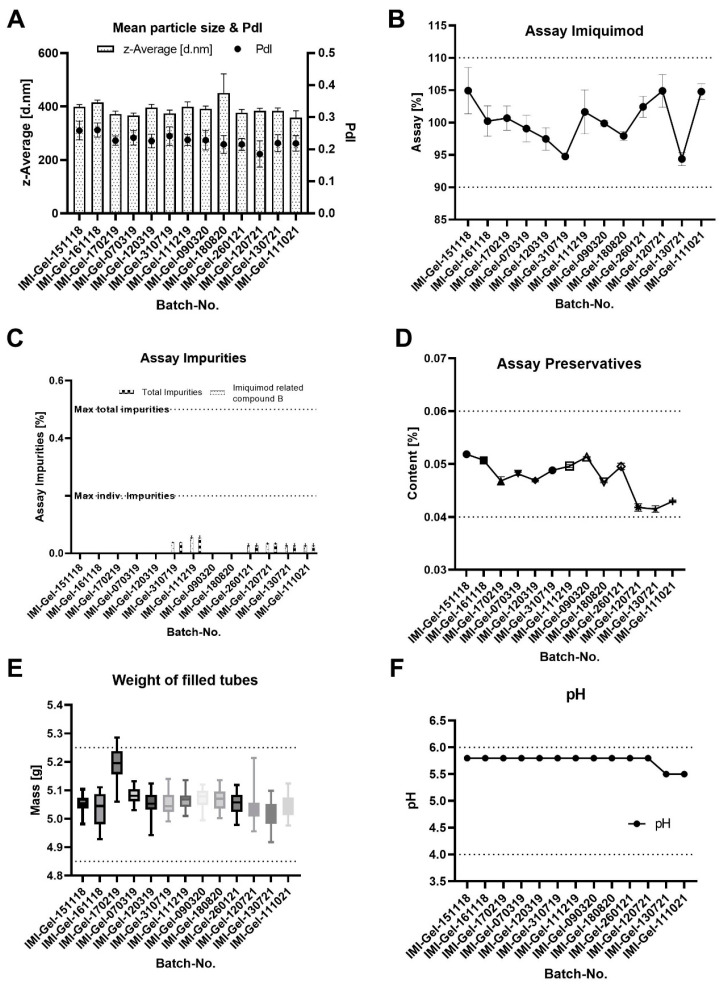
Batch-to-batch variability in the in-process control data for the mean particle size of the IMQ nanocrystals (**A**) and from QC data of the mean IMQ content (**B**), the level of impurities and degradants (**C**), the content of the preservatives methyl- and propylparaben shown as the total content (**D**), the weight of the filled tube units (**E**) and the pH (**F**). The data reveal minimal batch-to-batch variability in the manufactured batches. The grid lines represent the respective lower and upper specification limits.

Figure 4 shows the results obtained from the IPC and QC analyses. Given the trend analysis for the particle size (Figure 4A), the mean particle size and PdI of the IMQ nanoparticles (analyzed as IPCs during the manufacturing of IMP batches) is found to be slightly larger than predicted in the model but within the defined limits and with low batch-to-batch variability. The measured values of the mean particle-size range from 358.58 nm up to 451.4 nm, with a median of 384.4 nm and a 95% confidence interval of the median of 372.2 nm to 399.5 nm (see Table S2 for the full data). For the PdI, slightly larger values are observed in comparison to those in the model. The measured values range from 0.185 to 0.260, with a 95% confidence interval of 0.215–0.241 (see Table S3 for the full data).

The data from the content analysis of IMQ reveal minor fluctuations for the *mean content* between batches (ranging from 94.38% ± 1.02% to 104.93% ± 3.57%) with no out-of-specification (OOS) or out-of-trend (OOT) observations (see Figure 4B). All tested batches are within the defined acceptance criteria (mean content of 90% ≤ × ≤ 110% of the targeted dose of 5% (*w*/*w*) (see Table S4 for the full data)).

For the level of *impurities*, the data reveal that detected levels of impurities are observed for 6 out of the 13 manufactured batches. The detected levels are all significantly below the acceptance criteria (see Table S5 for the full data).

The data for the level of the *preservatives* reveal minor fluctuations in the mean preservative content, all within the acceptance criteria, where the latest manufactured batches reveal levels closer to the lower acceptance limit (see Table S6 for the full data).

Given the *weight of the filled tubes*, the data reveal that the box plot for the batch IMI-Gel-170219 exceeds the upper specification limit. An investigation of this OOS result revealed that 6 out of the 28 manufactured tubes are OOS. A root-cause analysis of this observation revealed that the mean weight of the primary packaging batch used for manufacturing exceeded the weight of previously used batches, while the filled mass of the formulation was within the targeted range of 3 g ± 15%. As a consequence, the affected primary packaging batch was withdrawn. The weight of the filled tubes for the remaining batches revealed no OOS or OOT results (see Table S7 for the full data).

The data for the *measured pH* of the formulation reveal a consistently measured pH for the tested batches. The measurements reveal a pH toward the upper specification limit (pH 6).

## 4. Discussion

### 4.1. Product Design

In general, a variety of formulation concepts for transportation of IMQ across the stratum corneum toward the target tissue are available, e.g., microemulsions (ME), microneedles (MN) and nanocrystals. The poor solubility of IMQ in most pharmaceutical oils (except for isostearic acid) and the ME concept to increase drug permeation across the skin outline the unsuitability of ME for the delivery of IMQ to the targeted upper skin layers. Additionally, MNs appear as unsuitable formulation concepts owing to their limited drug loading capacity (the required IMQ dose is 50 mg/g). Nanocrystals appear as suitable concepts matching the targeted decelerated IMQ skin permeation. Together with a suitable oil component, yielding an oil-in-water (O/W) emulsion with nanocrystalline IMQ particles, the further deceleration of IMQ skin permeation is achieved.

The thermodynamic instability in nanosuspensions and (nano)emulsions owing to the large surface free energy (∆G) of the nanosize disperse particles and oil droplets [22,23] requires the addition of stabilizing excipients to the product. The reduction in the large surface area in these systems appears (i) through the dissolution of incipient crystalline nuclei, causing particulate precipitation; (ii) through the agglomeration of small particles to reduce the surface free energy [22]; or (iii) by its leading to conventional phase separation phenomena in (nano)emulsions such as creaming, sedimentation, coalescence and flocculation [38]. Conventionally, stabilizing excipients such as *surfactants* (reducing the interfacial tension between the solid–liquid phase or liquid–liquid phase), *polymers* (to sterically stabilize the particles and droplets) and *crystal growth inhibitors* (to prevent Ostwald ripening) are added. The choice of the stabilizer is mostly empirical, but it must take into account interaction forces (hydrophilic, hydrophobic, hydrogen bonding or ionic forces) and a chain length long enough to create a steric barrier and the required HLB of the oil type. For IMI-Gel, the selected *surfactant* polysorbate 80 and *polymer* Carbopol were found to be suitable candidates to create and maintain nanocrystals while stabilizing the (nano)dispersed oil phase.

With respect to the type of the oil component, the strongest IMQ deceleration compound was identified as jojoba wax and selected for the product IMI-Gel. This observed strongest IMQ decelerated permeation is in line with previously reported decelerated diclofenac permeation across the skin for formulations containing jojoba wax [39]. This is supposed to be associated with the three-dimensional structure of jojoba wax.

The viscosity-enhancing agent is required for (i) the creation of a cream formulation of increased viscosity (~93 cP at a shear rate of 10 s^−1^ at 32 °C) for the prolonged residence time of the formulation on the skin while in parallel (ii) stabilizing the disperse IMQ particles and the oil phase. Given the available types of other macromolecular gelling agents, the synthetic gelling agent Carbopol is selected thanks to its good skin tolerability and maintenance of the gel structure over a broad pH range [40]. Other gelling agents, e.g., cellulose-based excipients, were previously identified as unsuitable for suspended IMQ [27]. As the neutralizing agent, sodium hydroxide as an inorganic base is selected because common reports suggest that nitrosamines (classified as probable human carcinogens) form when using neutralizing agents, such as triethanolamine.

### 4.2. Process Design and Identification of CMAs and CPPs

The designed process to manufacture IMI-Gel contains two major unit operations. (i) Wet media ball milling is selected for the creation of IMQ nanosuspensions as it is a widely used process to create nanosuspensions offering a robust, reproducible, scalable and solvent-free process. During milling, the suspension undergoes high-energy input where particles are broken down to the nanoscale by bead–bead collisions. Only a small fraction of the energy input is used to deform the particles [41], while most of the energy is converted into heat through dissipative mechanisms, e.g., viscous losses, inelastic bead–bead collisions and bead–wall collisions [42]. This leads to a rise in the temperature of the suspension, facilitating Ostwald ripening, the growth of the nanoparticles or surface modification and amorphization. Therefore, it is important to identify the material attributes of the drug substance that may be critical to a temperature rise, requiring temperature control by the adjustment of the process parameters of the milling process, as demonstrated by Guner et al. In their study, it was shown that the rate of the temperature increase in the suspension during a wet stirred media milling process depends on the selected process parameters of the stirrer speed, bead loading and bead size [43]. When selecting process conditions with lower energetic levels (the lowest stirrer speed, lowest bead loading and smallest beads), an overall temperature rise of 6 °C over 60 min was observed for the suspension [43]. In contrast, high energetic conditions (the fastest stirrer speed, largest bead loading and largest beads) caused a temperature jump from 18 °C to 45 °C in 2 min [43]. A helpful tool from a temperature rise perspective, designed in this study, is the thermal desirability score (TDS). The TDS rates a milling process on the basis of the temperature increase and the number of cycles—as excellent (corresponding to almost isothermal conditions), good, acceptable or poor [43]. Because the material attributes of IMQ, which has a high melting point (297–299 °C), features practically insolubility in water (also at elevated temperatures) and lacks known polymorphic forms with no change in the crystallinity of the drug before and after milling (see Figure S1), are uncritical for physicochemical instabilities, thanks to a temperature rise during milling, temperature control is not required.

(ii) HPH is chosen for the dispersion of jojoba wax because it offers a versatile and scalable process disrupting oils under high pressure to fine (nano)emulsions. During homogenization, high operational temperatures are generated, which are on the one hand advantageous as they decrease the viscosity of the formulation, enabling the passage of the formulation through the narrow gap of the homogenizer. On the other hand, high temperatures may lead to similar instabilities in nanosuspensions, as outlined above, and for oils sensitive to oxidation. For IMI-Gel, IMQ and jojoba wax are insensitive to high temperatures and oxidation. Therefore, a temperature rise during the process is uncritical.

### 4.3. Considerations and Evaluation of Selected CMAs

#### 4.3.1. IMQ Drug Substance

The criticality of the CMA of the *solubility* for IMQ is a function of the resulting formulation pH because IMQ is a weak base. The originator of the product Aldara found that increased aqueous solubilization for IMQ occurs when the pH is lowered, but with an incapability to solubilize the required 5% (*w*/*w*) IMQ dose even at an extremely acidic pH [27]. For the disperse systems present in IMI-Gel, the partial dissolution of the particles is undesirable as this may cause physical instabilities, e.g., Ostwald ripening in nanosuspensions. By defining and maintaining the lowest acceptable pH, of 4, solubility issues causing such instabilities can be avoided.

The CMA for the *occurrence of related substances* for IMQ is a function of the chemical stability of the IMQ drug substance. IMQ is reported to be a relatively stable drug (the originator of the Aldara formulation applied for a retest interval of 2 years after manufacturing [44]). In suspensions, degradation kinetics appear to be zero-order processes, where the large reservoir of solid drug particles leads to a constant concentration of the drug in the solution and, hence, a constant degradation rate for the drug molecules in the solution [45]. Thanks to the good chemical stability and suspended state of the particles, it is expected that the occurrence of related substances is uncritical. The results of the chemical stability investigations for IMQ confirmed these expectations [23].

#### 4.3.2. Stabilizing Excipients for IMQ Nanocrystals and Disperse Oil Phase

For polysorbate 80 as the stabilizing excipient for IMQ nanocrystals and disperse jojoba wax, the material attributes of *compatibility*, *HLB* and *concentration* reveal their criticality as they determine the stability of IMQ nanocrystals and the jojoba wax phase. *Compatibility* between IMQ and polysorbate 80 results when the surfactant ameliorates the increased wettability of the particles for the creation of nanoparticles, decreases interfacial tension and leads to sterical stabilization in the disperse solid nanocrystals, which is in turn a function of the used concentration. For the preparation of physically stable (nano)suspensions, it is recommended to select concentrations below the critical micellar concentration (CMC) to prevent instabilities such as aggregation or Ostwald ripening due to the solubilization of the particles. [46,47]. For IMQ, the used concentration of 0.9% (*w*/*w*) revealed the good compatibility and stabilization of the particles while exceeding the CMC (the CMC for polysorbate 80 has been reported to be from 13.4 ± 0.6 mg/L to 24.7 ± 1.4 mg/L [48]). However, even if small IMQ quantities may be solubilized at this concentration above the CMC, it was shown that the used concentration did not negatively affect the stability of the nanosuspension over 9 months of storage [22]. In contrast, the usage of concentrations above the CMC might be beneficial during processing, creating an excess of available surfactant for the rewetting of the newly created hydrophobic surface upon particle breakage [49]. Otherwise, particles might dewet or position themselves at an existing liquid–gas interface, leading to the formation of an undesirable Pickering foam during processing [49]. For the optimization of the surfactant concentration (preventing foam formation), a mathematical approach may be used to calculate the required surfactant/stabilizer concentration by calculating the specific surface area of the drug from the targeted particle-size distribution, followed by the theoretical surfactant quantity, estimated from the head area of the surfactant considered for drug stabilization [49]. Afterward, the actual quantity of surfactant can be calculated from the required volume of the suspension to achieve the required available extent of surfactant [49].

With regard to the *required HLB* for jojoba wax (~12.5), it is found that an HLB of 15, as used with polysorbate 80 in IMI-Gel, adequately stabilizes jojoba wax in the aqueous phase, even at a high concentration, of 45%. Because concentration optimization studies for polysorbate 80 for jojoba wax stabilization were not conducted but instead taken from the literature [30], it is obvious that the optimization of surfactant concentration or the usage of surfactant combinations will lead to the better/increased dispersity of jojoba wax.

#### 4.3.3. Oil Component

For the oil component of jojoba wax, its material attributes of *compatibility*, *required HLB* and *concentration* affect the permeation rate, stability and rheological properties of IMI-Gel. These attributes are significant contributors to the formulation performance and are therefore identified as CMAs. As outlined above, the optimal compatibility between the surfactant and the jojoba wax phase is a function of the required HLB. The used concentration influences the permeation rate. Therefore, the addition of high concentrations is required to maximize the deceleration of IMQ permeation.

#### 4.3.4. Gelling and Neutralizing Agent

The identified critical material attributes of the gelling and neutralizing agent of *compatibility*, *concentration*, *molecular weight* and *pKa* contribute to the resulting consistency of IMI-Gel, affecting the CQAs of the pH, cream stability and rheological properties and are therefore categorized as critical. *Compatibility* is given as long as the pH of the formulation is adjusted to a weak acidic or neutral pH to ensure the stability of IMQ nanocrystals and the sol-to-gel transitioning of Carbopol 974. The consistency of IMI-Gel is a function of the used *concentration* of the gelling agent and its molecular weight. The selection of the medium molecular weight Carbopol 974P as a gelling agent is a compromise between obtaining a formulation with a high viscosity (~30,000 cP at pH 7.5 at a concentration of 0.5% (*w*/*w*) [31]) to contribute to the stabilization of the disperse particulate and of the oil phase and to the prolonged residence time at the administration site while in parallel achieve the good spreadability of the formulation. Given the CMAs for the neutralizing agent, the attributes go hand in hand with the viscosity and pH of the formulation and therefore with the stability of the nanocrystals requiring stochiometric addition and pH control.

#### 4.3.5. Preservatives

The CMAs of the preservative’s *compatibility*, *concentration* and *log P* are relevant in that they determine the efficacy of the used preservatives and determine the microbiological stability of the formulation. While *concentration* is observed as uncritical, *compatibility* and *log P* revealed criticality, demonstrated in the incapability to fulfill the acceptance criteria of a conservation load test from Ph.Eur. 5.1.3. Parabens are known to show partitioning between hydrophilic and lipophilic phases and may be included in surfactant micelles, leading to a loss in efficacy [50]. Related to IMI-Gel, partitioning in the jojoba wax phase or inclusion in polysorbate 80 micelles might be the cause for the observed limited efficacy (for additional discussion, see Section 4.5), revealing that *log P* and *compatibility* are CMAs. As reported in the literature, parabens interact to a considerable extent with the polyoxyethylene macromolecules used in IMI-Gel (polysorbate 80) [50].

### 4.4. Evaluation of Potential CPPs

The milling process is a critical unit operation as it creates the IMQ nanocrystals, a fundamental component of the product. Consequently, the optimization of the process parameters, determining the resulting breakage kinetics and particle size, is required. This study focused on the evaluation and optimization of the critical process parameters of milling speed and milling time (as identified in the REM) as they determine the energy input and therefore the breakage rate of the drug particles, among other important process parameters [18]. In wet media milling, it is suggested that the breakup kinetics follow a first-order exponential decay, where the mean/median particle size approaches a limiting minimal particle-size value as a function of time [17,51,52,53]. The approximation of a lower particle-size plateau in prolonged milling can be explained by two major factors: (i) the formation of a dynamic equilibrium between particle breakage and the aggregation of the generated finer particles [46,54] and (ii) a decrease in the particle breakage rate thanks to the high strength of the nanoparticles, making them harder to break [42,55].

In this study, the evolution of the IMQ particle size during the wet media milling process follows an exponential decay (as outlined above), approaching a lower value of around 300 nm (although the grinding limit was not determined). In the generated DoE model, this is indicated by the fact that the linear and quadratic terms for the studied process parameters also have statistically significant impacts on the z-average, leading to a curved shape for the response-surface plot. Other studies in the literature also observed similar results with statistical significance for the milling time and the rotational speed on the particle size [56,57]. However, the application of a first-order kinetic model is known to have limitations, particularly that the early milling requires the elimination of early-milling kinetic data [42,53]. In the generated model, this is observed in the regression equation for the particle size, predicting an apparent initial mean IMQ particle size of 837.4 nm when the rotational speed and the milling time approach zero, although the initial particle size is much larger (*d*_10_ 4.96 µm, *d*_50_ 13.02 µm, *d*_90_ 26.39 µm).

The observed narrowing of the particle-size distribution over time (with the statistical significance for the linear term of the milling time) shows that an increased number of bead–bead collisions and drug particle compression is required in order to increase the extent of particle breakage and reduce the fraction of unmilled particles. When approaching the submicron (colloidal) size domain during milling, coarser particles continue to break (albeit slowly). In contrast, finer particles below a certain size limit will not break at all, or will break much slower, because they are harder to capture by beads and because the intensity/number of stressing events by the colliding beads may not be sufficiently high to further break them up [42,58]. Hence, this over time leads to a tightening of the particle-size distribution, as observed in this study.

Among the conducted pharmaceutical wet media milling studies in the literature, statistical approaches, including the response-surface method, as used in this study, are reported to be the most widely used [9]. Such empirical approaches allow a good understanding of the interaction effects and the selection of optimal experimental conditions, as observed in this study. The identified optimal process conditions lead to IMQ nanocrystal manufacturing at the targeted particle-size region with an acceptable accuracy between the predicted and observed values of the z-average and the PdI. Conducting additional experimental runs and studying other important process parameters, e.g., the number of beads and the bead size, will improve the predictability of the model between the process parameters, particle size and PdI.

A major limitation of statistical approaches, however, is their lack of including the underlying physics governing the milling process. One method to overcome this drawback is to use mechanistic modeling. In this approach, the complex physics occurring during wet media milling are modeled at different length scales (fluid mechanics, contact mechanics, bead collisions, particle capture, particle breakage, etc.), providing mechanistic-phenomenological modeling on a spectrum [9]. With, e.g., computational fluid dynamics (CFD), the discrete element method (DEM), population balance modeling (PBM) and microhydrodynamic modeling (MHD), the modeling of the wet media milling process may provide an improved quantitative fundamental understanding of the process, allowing for better process understanding and optimization. For example, Bilgili’s group adapted and refined a comprehensive MHD model first developed by Eskin [41] for the wet stirred media milling of pharmaceutical drug substances.

Despite the capability of the wet media milling process to produce nanosuspension for a broad variety of poorly water-soluble drugs, the process is associated with several drawbacks and knowledge gaps, particularly its processing-operational aspects: (i) the process is costly and energy intensive [59]; (ii) the process is time-consuming, with processing times ranging from hours to a day(s); (iii) the understanding of the stabilizer impact during milling beyond its stabilizing role is inadequate [9,42]; (iv) bead wear during processing leads to product contamination [18,58,60]; and (v) the scale-up of the process is mostly empirical [42,53]. When transferring between different equipment types for the manufacturing of nanosuspensions, challenges often occur because of differences in the geometry, mode of operation and power density of the mills, differently affecting the breakage kinetics and milling efficiency [18]. The milling behavior of a specific mill is characterized by the stress event intensity and the stress energy connected to each of the stress events [61]. Hence, the outcome of the comminution process is determined by the stress event frequency and intensity. While the optimized process showed an acceptable milling efficiency, an increase in milling efficiency may be required when moving to a larger device with a similar or different operating principle. Planetary ball mills, as used here, are more subject to early-phase development when the drug substance availability is limited, delivering small-scale suspension volumes, whereas wet stirred media mills are frequently used for pilot- and large-scale manufacturing, delivering larger suspension batch sizes at comparable or faster milling times [62]. The milling efficiency of the presented process may be increased by increasing the bead loading, as shown by Colombo et al. In their study, an increased milling efficiency for higher numbers of beads during the preparation of dexamethasone nanocrystals was observed [63]. Alternatively, the potential usage of various hydrodynamic parameters, e.g., the proposed milling intensity factor *F* by Afolabi et al. [17], may be useful for scale-up.

### 4.5. Formulation Performance Testing

For IMI-Gel, the essential evaluated attributes are the chemical stability ensuring the pharmacodynamic activity; the physical stability influencing the decelerated IMQ skin permeation as a function of the mean particle size; the stability of the emulsion and cream; and the microbiological stability (quality). As observed in chemical stability testing, the chemical stability of IMQ is not a critical attribute, indicated by the complete lack of loss in the drug product content and the complete lack of detectable levels of related substances, revealing that the pharmacodynamic activity is ensured over the shelf life of the product.

Physical stability for IMI-Gel refers to the physical stability of the IMQ nanocrystals in that they determine the decelerated IMQ permeation into the skin. As discussed in Section 4.3.2., the used surfactant polysorbate 80 leads to the good stabilization of the IMQ nanocrystals, revealing the physical stability of the nanosuspension over the shelf life and ensuring the targeted performance. In addition, physical stability refers to the structural stability of the product in that this determines the residence time at the site of action and the release of IMQ. Structural stability investigations by rheological analysis reveal good structural stability and no sensitivity toward shear or thermal stress (see [37]).

In terms of the microbiological stability, the stability tests reveal acceptable performance in terms of the inhibition of microbiological growth, but with the inability of the used surfactants to fulfil the test criteria of the monograph Ph.Eur. 5.1.3. on the efficacy of antimicrobial preservation. Thanks to the ability of the preservatives to inhibit microbiological growth, the regular authority agreed on a short in-use stability over 2 weeks after opening the primary packaging upon consultation. For further proceeding in drug product development, an evaluation of alternative preservatives, e.g., propylene glycol, isopropanol or ethanol, may yield favorable results fulfilling the test criteria of a conservation load test in Ph.Eur. 5.1.3. However, the compatibility with the IMQ nanocrystals and the oil phase needs to be demonstrated.

### 4.6. Control Strategy

The implementation of IPC and QC tests on the intermediate and final product(s) is intended to meet the targeted CQAs of the product, as defined in the acceptance criteria. While for all identified CQAs, the appropriate IPC or QC tests are installed, rheological quality control testing is excluded. The rationale for this decision is based on the fact that individual viscosity values or yield points do not represent material constants [64] and are therefore not considered as predictors of formulation performance. Instead, it is necessary to evaluate the structural stability of the formulation over its shelf life with the target attribute to maintain the structural stability over the shelf life of the formulation. This is indicated by no significant change in the flow properties and the ability to achieve structural recovery after the application of shear stress. The results from an organoleptic evaluation and, second, a rheological investigation using oscillatory three-interval-step tests have shown this attribute for IMI-Gel (see [37]).

Given the IPC and QC data, the application of the QbD concept enabled the development and manufacturing of high-quality IMPs with small batch-to-batch variability. For the mean particle size of the product, its observed small batch-to-batch variability outlines a robust and reproducible manufacturing process for the creation of nanocrystals close to the targeted range of ~400 nm, achieving the targeted product quality in terms of the particle size CQA. While for the batch IMI-Gel-180820, a higher mean particle size with a higher standard deviation than for the other batches is observed, the observation is not considered critical, because the resulting mean particle size is well within the acceptance criterion. In addition, the observed range of the particle size falls within the size range in which the particles migrate into the hair follicles.

For the content of IMQ, fluctuations are observed with a range of 10.15% (difference between the batch with lowest mean content and with the highest mean content). While this difference may appear significant, it is uncritical as values do not approach lower or upper specification limits, being well within the defined acceptance criterion of the product. In general, observing drug substance concentrations at the lower acceptance limit might be critical for substances with limited chemical stability (meaning that the concertation of the drug reaches a content below 90% during or at the end of the shelf life). For IMQ, however, achieving lower concentrations within the defined limits is not critical, thanks to the good chemical stability and limited degradation. Achieving higher dosing than that targeted may lead to overdosing and increased side effects, which are not observed with the manufactured batches.

As for the detected level of impurities, negligibly low measured levels underline the good chemical stability of IMQ.

Given the level of preservatives, the observed trend toward lower levels for the latest batches is considered uncritical because it was shown that the efficacy of the preservative is not concentration dependent. Manufactured batches should have at least a minimal content of 0.04% (that was found to inhibit microbiological growth), whereas larger concentrations do not positively affect microbiological growth

The QC test of the weight of the filled tubes is based on the requirement of minimum fill <755> included in the USP monograph imiquimod cream. The test is intended to test whether the patient has been provided with sufficient formulation. In terms of the weight of the filled tubes, it is important to provide the patient with sufficient formulation for the treatment of their AK lesions. Therefore, trends toward upper specification limits are not critical. During further proceeding, it is suitable to change the test by taking the mean weight of 10 packaging units and defining the total weight ± 15% from the detected mean weight of the packaging unit batch.

In terms of the pH, minor variabilities may be neglected because the material attributes potentially affected by the pH (such as the gelling agent Carbopol and the paraben preservatives) are compatible with a broad pH range. The trend of the QC outlines pH values toward the upper specification limit. This achieved pH is not considered critical, because pH values toward the neutral range are reported to enable the maintenance of a stable gel structure for Carbopol-based gels, prevent IMQ crystals from solubilization and are well within the pH range where the paraben preservatives are able to inhibit microbiological growth.

In terms of the microbiological evaluation of IMI-Gel, no trend is observed, in that all tested batches fulfill the test criteria of the chapter Ph.Eur. 2.6.12.

## 5. Conclusions

QbD is a systematic process for the development of high-quality medicines with a prospective design of the product in a QTPP, the identification of CQAs and the collection, evaluation and optimization of material attributes and process parameters to meet the predefined quality objectives, linking CMAs and CPPs to the CQAs. In this article, the concept of QbD is successfully employed for the development of a high-quality nanoparticulate IMQ emulsion gel formulation used as an IMP. The optimization of the product includes the design of a QTPP with the identification of CQAs and a discussion on the selected CMAs that allows for the selection of the excipients for the product. In addition, the optimization of the CPPs of milling time and milling speed by employing the statistical tool of DoE enabled the optimization of the process conditions for the manufacturing of IMQ nanocrystals in the target range of 300–400 nm at a minimal PdI. The results of the QbD process are depicted in the IPC and QC of the manufactured IMP batches, revealing small batch-to-batch variability and a product of high quality. Further studies on, e.g., material attributes, including other or additional surfactants at different concentrations or other process parameters in the milling process, e.g., number of beads and bead size or the optimization of the HPH process, may lead to a product of even higher quality.

## Figures and Tables

**Table 1:** Quality target product profile (QTPP) for the investigational medicinal product (IMP) IMI-Gel, where identification of CQAs are the stability, the particle size of IMQ, the rheological properties, the pH of the formulation, the content of the API, the homogeneity, the limits for impurities, the in vitro release profile and the microbiological limits.

QTPP Elements	Target	Criticality	Justification
Dosage form	Cream	----	Suitable dosage form for treatment of skin disease AK
Route of administration	topical	----	AK is a skin disease
Dosage strength	5% *w*/*w*	----	Similar strength to comparator Aldara product
Dosage design	Oil-in-water emulsion with dispersed IMQ nanocrystals and dispersed jojoba wax	----	---------
Stability	ICH Q1A: At least 6 months at room temperature	Yes	Quality requirement: maintain quality of product over shelf life
Drug product quality attributes	Physical attributes: ● Mean particle size of IMQ nanocrystals in the range of 300–400 nm, PdI ≤ 0.3 ● Rheological properties ● Oil droplet size < 10 µm ● pH 4.0–6.0	Yes Yes NO Yes	To meet applicable quality standards ● Target particle size to deliver IMQ into the hair follicles for slow and sustained absorption [12,15] ● Relevant for residence time of drug at administration site and stability [28] ● Ensure dispersity of the oil phase but droplet size not relevant for mode of action ● Relevant for efficacy of preservatives, prevention of pH-dependent solubilization of IMQ within the formulation (<1% solubilized at pH 4 [27]) and gel formation of the polyacrylate gel
	Identification	----	---------
	Assay: 90 ≤ × ≤ 110% of labeled amount based on USP 42 NF 37 IMQ Cream Monograph	Yes	Relevant for dosing uniformity, ensuring desired mode of action and limited adverse events owing to overdosing
	Homogeneity	Yes	Relevant for stability over treatment period
	Degradation products/ impurities based on USP 42 NF 37 IMQ Cream Monograph	Yes	Should be maintained below limits owing to relevance for efficacy and safety
	In vitro release profile	Yes	Relevant for slower permeation rate across the skin in comparison to reference Aldara product
	Content of preservatives: consult Ph.Eur. 5.1.3.	-----	-----
	Microbiological limits: consult Ph.Eur. 2.6.12. and Ph.Eur 5.1.4.	Yes	Must be maintained below specified limits in the pharmacopeia
Container closure system	Suitable for dosage form	----	Influences product stability, handling and acceptability/acceptance by the patient
Packaging integrity	Suitable over shelf life	----	Needed for stability, clinical effectiveness and safety

**Table 2:** Summary of identified potential CMAs with effects on the CQAs’ stability (including nanocrystal stability, cream stability, emulsion stability, microbiological stability and chemical stability), permeation rate, rheological properties, pH, content uniformity and particle-size distribution.

Formulation Components CMAs	CQAs
Drug substance IMQ Solubility Related substances	Stability of nanocrystals, permeation rate, pH, particle-size distribution Stability (degradants/impurities)
Surfactant polysorbate 80 Compatibility HLB Concentration	Content uniformity, stability (nanocrystals, emulsion), particle-size distribution Stability (of nanocrystals and emulsion) Stability (of nanocrystals and emulsion), particle-size distribution
Gelling agent Carbopol 974P Compatibility Concentration Molecular weight	pH, cream stability, rheological properties Rheological properties, cream stability Rheological properties, cream stability
Neutralizing agent sodium hydroxide Compatibility Concentration pKa	pH, rheological properties pH, rheological properties, stability (of nanocrystals, cream) pH, rheological properties, stability (of nanocrystals, cream)
Oil component jojoba wax Compatibility Required HLB Concentration	Permeation rate, stability (of emulsion) Permeation rate, stability (of emulsion) Rheological properties, permeation rate, stability (of emulsion)
Preservatives methyl- and propylparaben Compatibility concentration Log P	Stability (microbiological limits) Stability (microbiological limits) Stability (microbiological limits)

**Table 3:** Identification of CPPs (depicted in red in the REM and frequency histogram) affecting the CQAs of the particle-size distribution and the stability of the formulation, by factoring in the milling and the homogenization process steps.

Risk Assessment of Critical Process Parameters	CQAs
Wet media ball milling Milling time Rotational speed	Particle-size distribution Particle-size distribution
High-pressure homogenization Pressure	Stability (homogeneity)

**Table 4:** Analysis of variance (ANOVA) table revealing a statistically significant influence of the linear terms of the rotational speed (X_1_) and the milling time (X_2_), of the quadratic term of the rotational speed (X_1_)^2^ and for the term of 2FI (X_1_X_2_) on the response factor of the z-average and statistical significance for the linear term for the milling time X_2_ on the response factor PdI.

Source	Particle Size				Polydispersity Index (PdI)			
	Degrees of Freedom (DF)	Sequential Sum of Squares (Seq SS)	F Value	*p* Value	Degrees of Freedom (DF)	Sequential Sum of Squares (Seq SS)	F Value	*p* Value
Model X_1_ X_2_ (X_1_)^2^ (X_2_)^2^ X_1_·X_2_ Lack of fit test Pure error Total	5 1 1 1 1 1 3 17 25	97,143 33,507 53,373 6664 1310 2288 1529 4930 103,603	60.15 103.74 165.25 13.88 11.78 4.06 1.76 ---- ----	0.000 0.000 0.000 0.003 0.058 0.015 0.193 ----- -----	5 1 1 1 1 1 3 17 25	0.037890 0.000161 0.037074 0.000194 0.000333 0.000128 0.000188 0.015495 0.053574	9.66 0.21 47.28 0.04 0.42 0.16 0.07 ---- ----	0.000 0.655 0.000 0.835 0.522 0.690 0.976 ---- ----

**Table 5:** Analysis of variance (ANOVA) table after a reduction in the model revealing a statistically significant influence of the linear term the milling time (X_2_) on the PdI with an improved fit, indicated by the *p*-value of 0.986 for the lack of fit test.

Source	Polydispersity Index (PdI)			
	Degrees of Freedom (DF)	Sequential Sum of Squares (Seq SS)	F-Value	*p*-Value
Model X_1_ X_2_ Lack of fit test Pure error Total	2 1 1 6 17 25	0.037235 0.000161 0.037074 0.000843 0.015495 0.053574	26.21 0.23 52.19 0.15 ---- ----	0.000 0.638 0.000 0.986 ---- ----

## Data Availability

Supplementary data can be found under the Supplementary Materials file.

## Supplementary Materials

The following supporting information can be downloaded at: https://www.mdpi.com/article/10.3390/pharmaceutics15020514/s1, S1: HPLC Assay 1; S2: HPLC Assay 2; S3: HPLC Assay 3; Figure S1: Dynamic Scanning Calorimetry (DSC) Thermograms of as received crystals in red (left) and after milling in black (right); Table S1: Risk estimation matrix presenting initial risk assessment levels of individual material and process parameters: Low, low-risk parameter, medium, medium-risk parameter, High, high risk parameter; Table S2: Mean particle sizes of manufactured “IMI-Gel” batches measured per DLS with the Standard Deviation, the Median, 95 % Confidence Interval for the Median and the Coefficient of Variation for all measured data; Table S3: Mean PdI values of manufactured “IMI-Gel” batches measured per DLS with the Standard Deviation, the Median, 95 % Confidence Interval for the Median and the Coefficient of Variation for all measured data; Table S4: Mean content for manufactured “IMI-Gel” batches with respective Standard Deviation and Confidence Interval; Table S5: Level of impurities for the manufactured “IMI-Gel” batches with type of impurity (related compound A, B, C, D, E, or unknown); Table S6: Assay Preservatives for the manufactured “IMI-Gel” batches; Table S7: Minimum, 25% Percentile, Median, 75% Percentile and maximum weight of filled tubes from the manufactured “IMI-Gel” batches.

## Appendix A

**Table A1:** Face-centered central composite design (CCD) with observed responses for of the z-average and the PdI of the prepared IMQ nanosuspensions. The presented data from each condition (“run”) are arithmetical means of triplicate measurements (*n* = 3).

Run	Factors		Responses	
	Rotational Speed [rpm]	Milling Time [min]	Particle Size (z-Average) [d.nm]	Polydispersity Index (PdI)
1	650	60	404.4	0.264
2	450	150	357.6	0.149
3	450	240	318.2	0.132
4	450	150	353.9	0.183
5	250	240	388.5	0.204
6	450	60	460.5	0.274
7	450	240	367.7	0.202
8	450	150	391.6	0.199
9	650	150	327.5	0.136
10	450	150	356.2	0.189
11	650	240	292.8	0.126
12	450	150	387.2	0.175
13	450	150	360.3	0.211
14	250	150	422.1	0.204
15	450	150	363.1	0.219
16	250	60	523.2	0.308
17	650	240	305.2	0.167
18	450	60	430.7	0.231
19	450	150	376.3	0.206
20	250	60	589.7	0.237
21	250	240	384.2	0.094
22	650	60	398.5	0.244
23	450	150	361.9	0.21
24	650	150	342.9	0.127
25	250	150	437.9	0.189
26	450	150	381.7	0.21
