# Supplementary material for: Quality by Design (QbD) Approach for a Nanoparticulate Imiquimod Formulation as an Investigational Medicinal Product
Source: Pharmaceutics. 2023 Feb 3;15(2):514. doi: 10.3390/pharmaceutics15020514 (PMC9965879; doi:10.3390/pharmaceutics15020514)
Supplement: Supplementary file 1 [file pharmaceutics-15-00514-s001.zip › pharmaceutics-2104375-supplementary.pdf]

pharmaceutics

Supplementary Materials: Quality by Design (QbD) approach for a nanoparticulate imiquimod formulation as an investigational medicinal product

Jonas Pielenhofer, Sophie Luise Meiser, Karsten Gogoll, Anna Maria Ciciliani, Mark Denny, Michael Klak, Berenice M. Lang, Petra Staubach, Stephan Grabbe, Hansjörg Schild, Markus Radsak, Hilde Spahn-Langguth and Peter Langguth [1] *

S1. HPLC Assay 1:

Briefly, 200 mg of the formulation equivalent to 10 mg of IMQ was transferred into a 50 mL volumetric flask. 20 mL of a diluent (acetonitrile:water:phosphoric acid 250:750:10, v/v) was added to the flask. The sample solution was heated to 70 °C in a water bath for 5 min. After the samples were cooled to room temperature, the volume was filled up to the mark with the diluent, mixed and filtered through a Millex Nylon Syringe filter with a pore size of 0.45 µm and a diameter of 25 mm. For the reference standard, 100 mg of USP IMQ Reference Standard was transferred into a 50 mL volumetric flask and dissolved in the similar diluent as used for the sample. For the mobile phase, 2.0 g of heptane-1-sulphonic acid sodium salt was dissolved in 750 mL of water followed by addition of 1.5 mL of triethylamine and 250 mL of acetonitrile. The pH of the solution was adjusted to 2.7 ± 0.05 with phosphoric acid ≥85% and the mobile phase was degassed for 15 min in an ultrasonic bath. As the stationary phase, a Zorbax RX-C8 column of 150 mm length with a diameter of 4.6 mm and a particle size of 5 µm was used. 20 µL of the sample solution was injected into the system at a flow rate of 1.5 mL/min with a column temperature of 30 °C. The IMQ Peak appeared at ~11 min. The content of IMQ was calculated as the ratio of peak response for the sample peak divided by the peak response of the IMQ standard multiplied with the concentration of the IMQ reference standard solution divided by through the nominal concentration of the sample solution multiplied with 100. For QC analysis three samples were prepared as described above. The reference standard and samples were measured in triplicates.

S2. HPLC Assay 2:

All solvents used were of HPLC gradient grade. Briefly 1 g of IMI-Gel was transferred into a 20 mL volumetric flask followed by the addition of 1 mL of 2 M sulfuric acid solution. The flask was filled up to the mark with ethanol:water 90:10 diluent and heated to 60 °C for 5 minutes. Afterwards, the solution was quickly cooled to room temperature (RT) and kept at RT over night. For the analysis, the clear supernatant was used. For the HPLC analysis, the same system as described under 2.6.4. was used, equipped with the Zorbax RX-C8 of 150 mm length, 4.6 mm diameter and a 5 µm particle size. The mobile phase consisted of methanol:water (60:40) with the aqueous phase being adjusted to pH 2.2 ± 0.05 using phosphoric acid ≥ 85%. For the analysis, one sample per batch was prepared and analyzed in triplicates. Per sample, 20 µL of the sample solution was injected. As the calibration standards, 5 concentrations of 2.5 µg/mL, 5 µg/mL, 10 µg/mL, 20 µg/mL and 40 µg/mL of the reference standards were prepared from 1 mg/mL stock solutions of methyl- and propylparaben reference standard solutions and analyzed in triplicates. The retention times of the peaks methyl- and propylparaben were around 3 min and 5 min. The IMQ peak appeared immediately after the injection peak. The content of the preservatives was calculated by inserting the peak response of methyl- and propylparaben from the sample solutions into the regression equation obtained from the standards.

S3. HPLC Assay 3

Briefly, 400 mg of IMI-Gel was transferred into a 50 mL volumetric flask. 40 mL of a diluent of acetonitrile:water:phosphoric acid (650:350:1) was added to the sample followed by heating of the sample solution to 70 °C for 3 min with occasional stirring to suspend the formulation within the solution. Afterwards, the sample solution was cooled to room temperature, filled up to the mark with diluent and filtered through a Millex Nylon Syringe filter with a pore size of 0.45 µm and a diameter of 25 mm. For the IMQ reference standard solution, 50 mg of the USP IMQ reference standard was transferred accurately into a 50 mL volumetric flask and dissolved in the same diluent used for the sample solution. From this stock solution, a standard solution of 4 µg/mL was prepared by adding 20 µL of the stock solution to 4.98 mL of diluent. As the HPLC system, the same system as described under 2.6.4. and 2.6.5. was used. For the method, a gradient was used with three mobile phases. Mobile phase A was prepared by dissolving 1.0 g of heptane-1-sulphonic acid sodium salt, 0.8 g of sodium dodecyl sulfate and 1.0 g of dibasic potassium phosphate in 800 mL of water followed by addition of 200 mL of acetonitrile and mixing. Once at room temperature, the solution was adjusted with phosphoric acid ≥85 % to pH of 6.4 ± 0.05. Mobile phase B and C were prepared in analogous manner except for the ratio of water to acetonitrile being 400 ml to 600 mL for mobile phase B and 250 mL to 750 mL for mobile phase C. As the stationary phase, an Inertsil ODS-3 5 µm column with a length of 250 mm and an inner diameter of 4.6 mm was selected. For separation of IMQ and the related substances a gradient was used with an isocratic phase from 0-5 min composed of 80 % mobile phase A and 20 % mobile phase B, a gradient phase from 5-53 min from 80 % mobile phase A and 20 % mobile phase B to 40 % mobile phase A and 60 % mobile phase B, and isocratic phase of 100 % mobile phase C from 53-59 min and an equilibrium isocratic phase from 60-65 min of 80 % mobile phase A and 20 % mobile phase B. A flow rate of 1.2 mL/min at a column temperature of 30 °C was selected.

For QC analysis, one sample per batch was prepared. The reference standard solution and the sample solution were analyzed in triplicates.

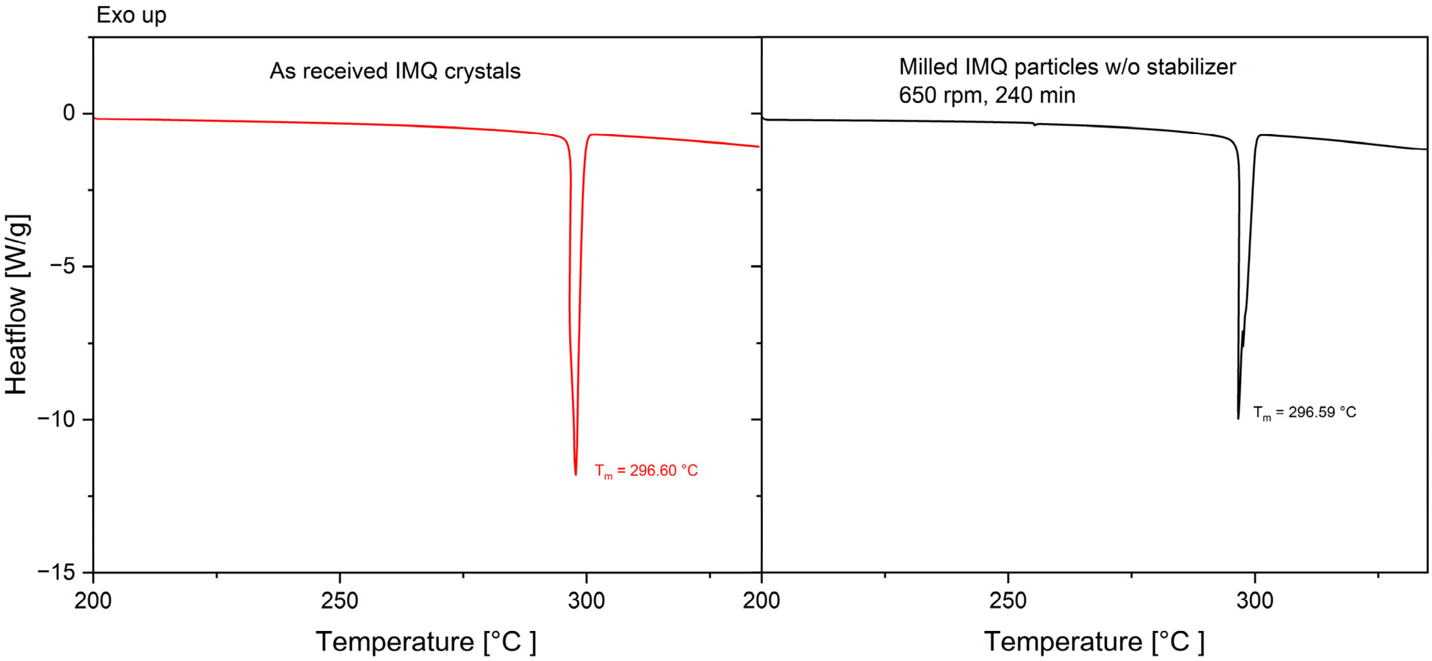

Figure S1. Dynamic Scanning Calorimetry (DSC) Thermograms of as received crystals in red (left) and after milling in black (right). The graphs show no change in the crystallinity of the drug after milling with a sharp melting point peak at a melting temperature T_m = 296.60 °C for the as received IMQ crystals and T_m = 296.50 °C for the milled IMQ particles

Supplement Table S1. Risk estimation matrix presenting initial risk assessment levels of individual material and process parameters: Low, low-risk parameter, medium, medium-risk parameter, High, high risk parameter

		Attribute	Content Uniformity	Particle size distribution	pH	Rheological properties	Permeation rate	Stability*
Raw Materials	IMQ	pKa	Low	Low	Low	Low	Low	Low
		Log P	Low	Low	Low	Low	Low	Medium
		Concentration	Low	Medium	Medium	Medium	Medium	Medium
		Solubility	Medium	High	High	Low	High	High
		Particle Size	Low	Medium	Low	Low	Low	Low
		Melting point	Low	Low	Low	Low	Low	Low
		Related substances	Medium	Low	Low	Low	Low	High
	Surfactant	Compatibility	High	High	Low	Low	Low	High
		HLB	Medium	Medium	Low	Medium	Low	High
		Concentration	Medium	Medium	Low	Medium	Low	High
		Log P	Medium	Medium	Low	Low	Low	Medium
	Gelling agent	Compatibility	Low	Low	High	High	Low	High
		Concentration	Low	Low	Medium	High	Medium	High
		Molecular weight	Low	Low	Low	High	Medium	High
		pKa	Low	Low	Medium	Medium	Low	Medium
	Neutralizing agent	Compatibility	Low	Low	High	High	Low	Medium
		Concentration	Low	Low	High	High	Medium	High
		pKa	Low	Low	High	High	Medium	High
	Oil component	Compatibility	Low	Low	Low	Medium	High	High
		Required HLB	Low	Low	Low	Medium	High	High
		Concentration	Low	Low	Low	High	High	High
		Viscosity	Low	Low	Low	Medium	Medium	Medium
	Preservatives	Compatibility	Low	Low	Low	Low	Low	High
		Concentration	Low	Low	Low	Low	Low	High
		Log P	Low	Low	Low	Low	Low	High
Process Parameters	Wet Media ball milling	Milling ball size	Low	Medium	Low	Low	Low	Medium
		Temperature	Low	Low	Low	Low	Low	Low
		Ratio Drug to Milling balls	Low	Medium	Low	Low	Low	Medium
		Rotational speed	Low	High	Low	Low	Low	High
		Milling time	Low	High	Low	Low	Low	High
	High Pressure Homogenization	Temperature	Low	Low	Low	Low	Low	Low
		Number of Cycles	Medium	Medium	Low	Medium	Low	Medium
		Pressure	Medium	Medium	Low	Medium	Low	High
	Gel formation	Viscosity	Low	Low	Low	Medium	Low	High
		Concentration	Low	Low	Low	Medium	Low	Medium
	Filling	Time	Low	Low	Low	Medium	Low	Medium
		Shear stress	Low	Low	Low	Medium	Low	Medium
		Amount	Low	Low	Low	Low	Low	Low

*Stability includes the CQAs Homogeneity, Impurities/Degradants and microbiological limits

Supplement Table S2. Mean particle sizes of manufactured „IMI-Gel" batches measured per DLS with the Standard Deviation, the Median, 95 % Confidence Interval for the Median and the Coefficient of Variation for all measured data

Batch-No.	Mean z-Average [d.nm]	Standard Deviation [d.nm]	N	Acceptance criterion
IMI-Gel-151118	399.22	8.74	18	
IMI-Gel-161118	415.37	8.75	18	
IMI-Gel-170219	372.24	10.77	18	
IMI-Gel-070319	366.85	8.82	18	
IMI-Gel-120319	396.31	11.84	18	
IMI-Gel-310719	374.04	12.85	18	
IMI-Gel-111219	399.51	18.29	18	z-Average 400 ± 200 nm
IMI-Gel-090320	391.76	9.82	18	
IMI-Gel-180820	451.41	70.93	18	
IMI-Gel-260121	377.39	11.64	18	
IMI-Gel-120721	384.43	9.46	18	
IMI-Gel-130721	383.54	11.17	18	
IMI-Gel-111021	358.58	25.71	18	
Median	384.4	--------	-------	--------
Range	92.83	--------	-------	--------
95 % Confidence interval for the Median	372.2 – 399.5	--------	-------	--------
Coefficient of Variation [%]	6.17	--------	-------	--------

Supplement Table S3. Mean PdI values of manufactured „IMI-Gel" batches measured per DLS with the Standard Deviation, the Median, 95 % Confidence Interval for the Median and the Coefficient of Variation for all measured data

Batch-No.	PdI	Standard Deviation	N	Acceptance criterion
IMI-Gel-151118	0.259	0.029	18	
IMI-Gel-161118	0.260	0.022	18	
IMI-Gel-170219	0.227	0.015	18	
IMI-Gel-070319	0.236	0.022	18	
IMI-Gel-120319	0.226	0.021	18	
IMI-Gel-310719	0.241	0.029	18	
IMI-Gel-111219	0.229	0.017	18	PdI < 0.3
IMI-Gel-090320	0.229	0.030	18	
IMI-Gel-180820	0.215	0.028	18	
IMI-Gel-260121	0.215	0.019	18	
IMI-Gel-120721	0.185	0.041	18	
IMI-Gel-130721	0.219	0.026	18	
IMI-Gel-111021	0.219	0.024	18	
Median	0.227	--------	--------	--------
Range	0.075	--------	--------	--------
95 % Confidence interval for the Median	0.215 - 0.241	--------	--------	--------
Coefficient of Variation [%]	6.17	--------	--------	

Table S4. Mean content for manufactured „IMI-Gel" batches with respective Standard Deviation and Confidence Interval

Batch-No.	Mean content [%]	Standard Deviation [%]	95 % Confidence Interval [%]	N	Acceptance criterion
IMI-Gel-151118	104.93	3.58	102.2-107.70	9	90 ≤ x ≤ 110% of 5 % (w/w)
IMI-Gel-161118	100.23	2.33	98.28-102.20	8*	
IMI-Gel-170219	100.68	1.87	99.25-102.10	9	
IMI-Gel-070319	99.07	2.06	97.48-100.70	9	
IMI-Gel-120319	97.46	1.74	96.12-98.80	9	
IMI-Gel-310719	94.75	0.35	94.49-95.02	9	
IMI-Gel-111219	101.64	3.41	99.02-104.3	9	
IMI-Gel-090320	99.87	0.42	99.55-100.2	9	
IMI-Gel-180820	97.94	0.70	97.40-98.48	9	
IMI-Gel-260121	102.43	1.62	101.2-103.7	9	
IMI-Gel-120721	104.89	2.53	102.9-106.8	9	
IMI-Gel-130721	94.38	1.02	93.60-95.17	9	
IMI-Gel-111021	104.77	1.21	103.8-105.7	9	

* one was identified as a statistical significant outlier in an outlier test with a $p < 0.05$ using Grubb's outlier test and excluded from analysis

Table S5. Level of impurities for the manufactured „IMI-Gel" batches with type of impurity (related compound A, B, C, D, E, or unknown)

Batch-No.	Detected Impurity type	Level [%]	Total level of Impurities [%]	N	Acceptance criterion
IMI-Gel-151118	-----	< 0.1	< 0.1	3	Individual level of impurities ≤ 0.2 %<br>Unknown impurities ≤ 0.1 %<br>Total level of impurities ≤ 0.5 %
IMI-Gel-161118	-----	< 0.1	< 0.1	3	
IMI-Gel-170219	-----	< 0.1	< 0.1	3	
IMI-Gel-070319	-----	< 0.1	< 0.1	3	
IMI-Gel-120319	-----	< 0.1	< 0.1	3	
IMI-Gel-310719	B	0.038	0.038	3	
IMI-Gel-111219	B	0.056	0.056	3	
IMI-Gel-090320	-----	< 0.1	< 0.1	3	
IMI-Gel-180820	-----	< 0.1	< 0.1	3	
IMI-Gel-260121	B	0.028	0.028	3	
IMI-Gel-120721	B	0.034	0.034	3	
IMI-Gel-130721	B	0.028	0.028	3	
IMI-Gel-111021	B	0.028	0.028	3	

Table S6. Assay Preservatives for the manufactured „IMI-Gel" batches

Batch-No.	Assay Preservatives [%]	Standard Deviation [%]	N	Acceptance criterion
IMI-Gel-151118	0.052	0.0002	3	
IMI-Gel-161118	0.051	0.0001	3	
IMI-Gel-170219	0.047	0.0008	3	
IMI-Gel-070319	0.048	0.0003	3	
IMI-Gel-120319	0.047	0.0003	3	
IMI-Gel-310719	0.049	0.0002	3	
IMI-Gel-111219	0.050	0.0003	3	0.04 – 0.06 % (w/w)
IMI-Gel-090320	0.051	0.0001	3	
IMI-Gel-180820	0.047	0.0002	3	
IMI-Gel-260121	0.050	0.0006	3	
IMI-Gel-120721	0.042	0.0007	3	
IMI-Gel-130721	0.042	0.0006	3	
IMI-Gel-111021	0.043	0.0003	3	

Table S7. Minimum, 25% Percentile, Median, 75% Percentile and maximum weight of filled tubes from the manufactured „IMI-Gel" batches

Batch-No.	Minimum [g]	25% Percentile [g]	Median [g]	75% Percentile [g]	Maximum [g]	N	Acceptance criterion
IMI-Gel-151118	4.982	5.036	5.052	5.074	5.104	28	
IMI-Gel-161118	4.928	4.981	5.045	5.087	5.110	28	
IMI-Gel-170219	5.060	5.156	5.195	5.238	5.285	28	
IMI-Gel-070319	5.030	5.061	5.080	5.104	5.131	24	
IMI-Gel-120319	4.943	5.032	5.053	5.084	5.124	30	
IMI-Gel-310719	4.991	5.023	5.045	5.084	5.140	28	
IMI-Gel-111219	5.010	5.041	5.068	5.081	5.135	27	Weight of filled tubes: 5 g ± 15 %
IMI-Gel-090320	4.995	5.048	5.079	5.099	5.120	27	
IMI-Gel-180820	5.002	5.035	5.070	5.096	5.135	30	
IMI-Gel-260121	4.979	5.025	5.058	5.084	5.118	28	
IMI-Gel-120721	4.956	5.007	5.043	5.056	5.213	30	
IMI-Gel-130721	4.917	4.982	5.004	5.052	5.098	31	
IMI-Gel-111021	4.976	5.012	5.032	5.075	5.124	26	
